# If in doubt, sit them out: concussion management in the community

**DOI:** 10.3399/BJGP.2026.0408

**Published:** 2026-10-01

**Authors:** Christian Mallen, Neil Heron, Clare Pheasey, Tom Crick, Naomi D Deakin, Peter Hutchinson

**Affiliations:** 1 Faculty of Medicine and Health Sciences, Keele University, Keele, UK; 2 Centre for Public Health, School of Medicine, Queen’s University Belfast, Belfast, UK; 3 Institute of Sport, Keele University, Keele, UK; 4 Faculty of Arts, Law and Social Sciences, University of Bristol, Bristol, UK; 5 Department for Culture, Media and Sport, UK Government, London, UK; 6 Department of Neurosurgery, Cambridge University Hospitals NHS Foundation Trust, Cambridge, UK; 7 East of England Neurosurgery Network, Cambridge University Hospitals NHS Foundation Trust, Cambridge, UK; 8 Department of Clinical Neurosciences, University of Cambridge, Addenbrooke’s Hospital, Cambridge, UK

## Clinical scenario

It is a familiar situation in general practice; 17:20 on a Thursday afternoon and you take a call from a parent. The school has asked them to make contact because their child sustained a head injury during PE. They have ‘a bit of headache’, and their teacher would like them ‘checked out’. After this telephone triage, you see the patient and parent in clinic. The physical examination is unremarkable, with the exception that they feel a bit tired and still report a headache. You are left with a familiar dilemma: refer to an already stretched emergency department, knowing there is a >5 hour wait, or manage conservatively with written information and instructions to make contact should anything change?

## Mild traumatic brain injury

In England and Wales ~1.4 million patients per year attend hospital following head injury,^1^ with >90% of these being classed as ‘mild’. Grassroots sports are the second most common cause of mild traumatic brain injury (mTBI) in this setting,^2^ yet management remains suboptimal and is inconsistent across settings.^3,4^ The reasons for this are multifactorial, including inconsistent identification, injury non-disclosure, lack of guideline awareness, inadequate advice and if given treatment, premature discharge from available services, with significant variability in care pathways nationally. These inconsistencies risk both under-recognition and premature return to activity, potentially exacerbating symptoms and increasing the likelihood of prolonged recovery.

*”… there is low public awareness of the ‘If in doubt, sit them out: UK concussion guidelines for non-elite (grassroots) sport’ …”*

## UK concussion guidelines for non-elite (grassroots) sport

To address barriers to care and increase awareness, the UK Government published new guidance in 2024, *If in doubt, sit them out: UK concussion guidelines for non-elite (grassroots) sport*.^5^ This provides a simplified framework, highlighting key messages and giving consistent guidance to various stakeholders; three key messages are described:

1. Recognise the signs of concussion (Box 1);
2. Remove anyone suspected of being concussed immediately;
3. Return safely to daily activity, education/work and, ultimately, sport.

**Box 1.** Red flags requiring urgent medical assessment^a^

Any loss of consciousness	Seizure/convulsion or limb twitching or lying rigid/motionless due to muscle spasm
Deteriorating consciousness	Severe or increasing headache
Amnesia for events before or after the injury	Severe neck pain
Increasing confusion or irritability	Suspicion of skull fracture
Repeated vomiting	Previous history of brain surgery or bleeding disorder
Unusual behaviour change	Current anti-coagulation
New neurological deficit including difficulties understanding speaking, reading or writing, decreased sensation, loss of balance, weakness, double vision	Current drug or alcohol intoxication

^a^Source: UK concussion guidelines for non-elite (grassroots) sport, 2024^2,5^

## Challenges for GPs

This creates a practical challenge for GPs; how to balance limited consultation time with diagnostic uncertainty while ensuring safe and appropriate management.

### Recognition of challenges

Better recognition of concussion is critical. While symptoms often appear immediately, they may be delayed, becoming evident 24–48 hours after the head impact. Symptoms include disorientation, headache, visual disturbance, nausea/vomiting, fatigue, and confusion, with such non-specific symptoms complicating the clinical diagnosis.

### Assessment limitations

Assessment instruments, such as pitch-side SCAT6^6^ or office-based SCOAT6^7^ can be used, but their application to primary care settings is limited,^8^ due to the duration of assessment and necessary demographic adjustments. Most UK GP appointments are allocated 10 minutes, while SCAT6 can be performed within this timeframe, completion of the more detailed SCOAT6 is not possible. Also, standard assessment tools are relevant to those aged ≥13 years, with Child SCAT6^9^ and Child SCOAT6^10^ validated only in those >8 years old. For this freely available assessment, there are currently no validated tools for children <8 years, and limited validation for languages spoken in the UK beyond English.

### Recovery and follow-up

For most people with a concussion, symptoms will resolve within 2–4 weeks, However, patients require guidance and management on returning to life, education, work, and then sport. Also, 10–20% of cases will persist beyond 4 weeks, often requiring specialist healthcare input that may not be available locally. Relevant NICE guidelines^11^ advise referring patients with persistent symptoms post-concussion to specialists with relevant expertise, including neurologists, rehabilitation, sport and exercise medicine professionals, with the grassroots concussion guidance indicating review by a GP if symptoms have not resolved in 28 days.

## Next steps to improve concussion management in the community

To help address the significant health burden of concussion in the community, a number of steps need to be taken. To increase awareness, high-quality concussion education should be provided to healthcare workers and the public, specifically detailing its management and the potential morbidity. To bridge the gap between established primary care and limited emerging specialist clinics, we propose a network of community concussion clinics to support patients and healthcare workers through concussion management, as well as managing people with persistent^11^ symptoms beyond 4 weeks (defined as post-concussion syndrome). Over the past decade, outpatient concussion clinics, predominantly focused on sport, have been developed in some areas of England, with the most established NHS services in Cambridge and Birmingham. Both services are well supported by a varied multidisciplinary team encompassing surgical and medical specialties, as well as colleagues in nursing, physiotherapy, and clinical neuropsychology. Following the UK Concussion Conference (London, 2025), and in collaboration with the NIHR HealthTech Research Centre in Brain Injury, there is significant support for a national network of similar specialist services across England and the devolved nations. We believe that these clinics should be developed with patients and their broader support network (including the education sector), and include input from primary care, community bodies (such as sports clubs) and third sector partners. Together, we aim to identify and enact solutions at community level to address the common issues that patients with concussion face.

## Next steps for concussion and research in primary care

Areas of concussion research that require development in community healthcare include prediction of prolonged symptoms, provision of validated tools and implementation of novel biomarkers.

### Prediction

In the first instance, accurate prediction of children and adolescents who will develop prolonged post-concussion symptoms would allow better targeting of early interventions to this young and vulnerable group.^12^

### Assessment tools and digital innovation

Specific to the time constrained model of primary care appointments, we also support development and validation of rapid, office-based concussion assessment tools, in addition to exploration of digital options for remote monitoring and management.^13^

### Biomarkers

Future work could also investigate concussion biomarkers and their application to general practice, supporting both diagnosis and prognosis across the broad spectrum of our patient population.

“*Improving outcomes requires greater awareness, practical tools tailored to the realities of general practice, and the development of community-based concussion services.”*

## Conclusion

Concussion is a common injury within the community. Despite this, there is low public awareness of the *If in doubt, sit them out: UK concussion guidelines for non-elite (grassroots) sport* ^5^, and patchy implementation across primary care and wider community sports infrastructure. Closing this gap will need the same cross government collaboration that delivered the guidance itself — bringing together primary care, NHS bodies, and Department for Culture, Media and Sport-convened structures. Improving outcomes requires greater awareness, practical tools tailored to the realities of general practice, and the development of community-based concussion services. Within the context of developing specialist concussion clinics, we have identified a clear need for community concussion clinics and strongly support formal collaboration with the NIHR HealthTech Research Centre in Brain Injury, which has established a new working group to refine clinical guidance and prioritise research.

Through coordinated action across physical and mental healthcare, education and research, there is a clear opportunity to transform concussion care at a population level. This is particularly relevant for services for children and adolescents, which represent an unmet need. We propose partnership working with input from regional health leaders to develop the required local resources. As this model is grounded in the English NHS landscape, equivalent work — reflecting the guidelines’ four-nations basis — should be catalysed in Scotland, Wales, and Northern Ireland to ensure UK-wide implementation. This should be paired with early, sustained, and meaningful co-creation with patients, their support network, and non-statutory partners, including charitable bodies.
